# Age-Dependent Changes in the Sphingolipid Composition of Mouse CD4^+^ T Cell Membranes and Immune Synapses Implicate Glucosylceramides in Age-Related T Cell Dysfunction

**DOI:** 10.1371/journal.pone.0047650

**Published:** 2012-10-26

**Authors:** Alberto Molano, Zhaofeng Huang, Melissa G. Marko, Angelo Azzi, Dayong Wu, Elaine Wang, Samuel L. Kelly, Alfred H. Merrill, Stephen C. Bunnell, Simin Nikbin Meydani

**Affiliations:** 1 Jean Mayer USDA Human Nutrition Research Center on Aging, Tufts University, Boston, Massachusetts, United States of America; 2 The Parker H. Petit Institute for Bioengineering and Bioscience, Georgia Institute of Technology, Atlanta, Georgia, United States of America; 3 Department of Pathology, Sackler Graduate School of Biomedical Sciences, Tufts University, Boston, Massachusetts, United States of America; Laboratory of Neuroendocrine-Immunology, Pennington Biomedical Research Center, United States of America

## Abstract

To determine whether changes in sphingolipid composition are associated with age-related immune dysfunction, we analyzed the core sphingolipidome (i.e., all of the metabolites through the first headgroup additions) of young and aged CD4^+^ T cells. Since sphingolipids influence the biophysical properties of membranes, we evaluated the compositions of immune synapse (IS) and non-IS fractions prepared by magnetic immuno-isolation. Broadly, increased amounts of sphingomyelins, dihydrosphingomyelins and ceramides were found in aged CD4^+^ T cells. After normalizing for total sphingolipid content, a statistically significant decrease in the molar fraction of glucosylceramides was evident in both the non-IS and IS fractions of aged T cells. This change was balanced by less dramatic increases in the molar fractions of sphingomyelins and dihydrosphingomyelins in aged CD4^+^ T cells. *In vitro*, the direct or enzymatic enhancement of ceramide levels decreased CD4^+^ T cell proliferation without regard for the age of the responding T cells. In contrast, the *in vitro* inhibition of glucosylceramidase preferentially increased the proliferation of aged CD4^+^ T cells. These results suggest that reductions in glucosylceramide abundance contribute to age-related impairments in CD4^+^ T cell function.

## Introduction

Genetic studies in yeast and other model organisms have implicated several pathways in the aging process. Genes involved in the control of metabolism, stress resistance, chromatin-dependent gene regulation and genome stability are frequently selected in mutational studies of longevity regulation [1], [2]. Products of sphingolipid metabolism, such as ceramides (Cer), modulate many of these pathways [3]. Thus, alterations in sphingolipid metabolism may contribute to dysfunctions associated with age, and may provide attractive targets for preventive/therapeutic intervention.

Sphingolipid metabolites play roles in both stress and aging. In *Saccharomyces cerevisiae*, the LAG1 gene (longevity assurance gene 1) encodes a component of the enzyme ceramide synthase. Although simultaneous deletion of both LAG1 and a homologous gene, LAC1, is lethal, the deletion of LAG1 alone extends the lifespan of *S. cerevisiae*, implicating Cer metabolites in aging [4]. Heat stress triggers enhanced *de novo* sphingolipid synthesis in yeast [5], and different forms of stress appear to constitute a frequent cause of elevated sphingolipid production in more complex organisms as well [3]. Similarly, DNA intercalating agents and other inducers of genotoxic stress, such as gamma irradiation, often result in elevated endogenous Cer levels [6]. Cer, in turn, modulate signaling pathways that operate in response to these stressful insults. These pathways control basic cellular processes, such as cell cycle progression, which is usually interrupted to allow certain cellular repair mechanisms to operate, or apoptosis, which may be triggered if the stressful insult overwhelms the cell's capacity for auto-repair [7], [8].

Cer are at a central “hub” of sphingolipid metabolism, where these lipid backbones are made *de novo* or by recycling of pre-existing sphingoid bases, then partition into different categories of more complex sphingolipids, e.g., sphingomyelins (SM) vs glycosphingolipids (GSL), or are turned over [3]. The physiological levels of these sphingolipids differ by several orders of magnitude, reflecting the divergent, and sometimes opposing, functions of these compounds [3], [9]. SM are typically the most abundant, consistent with their widely appreciated roles in membrane structure and the formation of ordered lipid microdomains [10]. The steady state levels of Cer are typically 5 to 10 times lower than SM, perhaps reflecting their status as metabolic intermediates and their involvement in stress-related signaling, including the induction of apoptosis. Glucosylceramide (Glc-Cer) levels are roughly comparable to those of Cer and also function as intermediates for biosynthesis of downstream products (more complex GSL); however, in contrast to Cer, Glc-Cer exhibit pro-proliferative effects in several model systems [11], [12], [13], [14]. Somewhat amazingly, mammals have been discovered recently to produce small amounts of (dihydro) ceramides (DH-Cer) that lack the hydroxyl-group at position 1, therefore, cannot be metabolized to more complex compounds [15]. These are intriguing compounds nonetheless because they have been found to bind to CD1b, and thus might affect immune function [16]. At the other end of the metabolic pathway, the ceramide metabolite sphingosine 1-phosphate (SoP), which can be 1000-fold less abundant than SM, is a chemoattractant for immune cells [3], and promotes proliferation, survival, and inhibition of apoptosis [17].

Importantly, the efficacy of the mammalian immune system declines with age [18]. In particular, several age-associated defects have been documented in the activation of CD4^+^ T cell by antigen. These include defects in the assembly of the immunological synapse joining the T cell to the antigen presenting cells [19], alterations in the assembly of signal transducing complexes within the IS [20], and reductions in the production of the pro-proliferative cytokine IL-2 and in T cell proliferation [21]. These molecular defects may contribute to the increased morbidity and mortality from infectious and neoplastic disease in the elderly.

Age-related alterations in sphingolipid metabolism could influence CD4^+^ T cell function in several ways. On one hand, global changes in the abundances of sphingolipid metabolites could influence T cell function via direct impacts on the biochemical pathways regulating T cell survival and proliferation. On the other hand, changes in the total or relative abundances of specific sphingolipids could perturb the assembly of T cell receptor (TCR)-proximal membrane microdomains implicated in T cell activation. Consistent with the latter hypothesis, magnetically isolated membrane microdomains nucleated by the TCR display increased levels of cholesterol, saturated phospholipids, and sphingolipids when compared to either total membranes or analogous microdomains nucleated by the transferrin receptor [22]. In this report, we evaluated age-associated changes in the core sphingolipidome (i.e., all of the metabolites through the first headgroup additions) of CD4^+^ T cells. In addition, we evaluated the sphingolipid composition of magnetically isolated signaling complexes derived from the immunological synapse. These studies employed state of the art sphingolipidomic methods, and were able to reveal changes in the absolute and relative abundances of specific sphingolipid species with greater precision than has previously been achieved [23], [24]. Our results highlight the potential contribution of altered sphingolipid composition to immune dysfunction in aged CD4^+^ T cells.

## Materials and Methods

### Animals

Young (4–6 month-old) and old (24–26 month-old) specific pathogen-free male C57BL/6 mice obtained from the National Institute on Aging colonies at Charles River Laboratories (Kingston, NY) were fed autoclaved Harlan Teklad 7012 mouse chow (Indianapolis, Indiana) and water, ad libitum. Mice were housed in filtered cages and maintained at a constant temperature (23°C) with a 12-hour light-dark cycle. Mice were euthanized via CO_2_ asphyxiation and spleens were aseptically removed and placed in sterile, endotoxin-free RPMI 1640 (BioWhittaker, Walkersville, MD) medium supplemented with 25 mM HEPES, 2 mM glutamine, 100 U/ml penicillin, and 100 µg/ml streptomycin (all from Life Technologies, Grand Island, NY; complete RPMI). Animal research was conducted in conformity with the Public Health Service policy on humane care and use of laboratory animals. The animal protocol for the current study (MS-55: Age-related changes in the proteome and lipidome of the immunological synapse) was approved by the Animal Care and Use Committee of the JMUSDA Human Nutrition Research Center on Aging at Tufts University and all the procedures were conducted following the approved protocol and in compliance with the NIH Guidelines for the Care and Use of Laboratory Animals. Mice were euthanized by CO_2_ asphyxiation followed by cervical dislocation and tissues were collected post-mortem. Mice exhibiting tumors, splenomegaly, grossly visible skin lesions, or significant pathology were excluded from the study. Through the surveillance program at the comparative biology unit, mice were continuously screened for specific pathogens.

### Purification of CD4^+^ spleen cells by negative selection

Single cell suspensions were prepared by gently disrupting spleens between two sterile frosted glass slides. Red blood cells were lysed using a hemolytic ammonium chloride-based reagent (Sigma; St. Louis, MO). Splenocytes depleted of red blood cells were washed with RPMI complete medium and resuspended in degassed buffer composed of 1X PBS, 2 mM EDTA, and 0.5% BSA (Sigma, St. Louis, MO). CD4^+^ T cells were purified using CD4^+^ T cell isolation kits II and LS columns, both from Miltenyi-Biotec (Auburn, CA), following the manufacturer's instructions. To assess purity, negatively selected cells were stained with APC-conjugated anti-CD4 along with other lymphocyte markers, such as anti-CD8 and anti-CD19 (from BD PharMingen; San Diego, CA), and analyzed on an Accuri C6 flow cytometer (Ann Arbor, MI) achieving at least 95–98% purity.

### Immune synapse isolation

Superparamagnetic polysterene M-500 Dynabeads designed for subcellular fractionation (Dynal; Lake Success, NY) were coupled with anti-CD3ε (145-2C11) and anti-CD4 (GK 1.5) monoclonal antibodies, as well as with recombinant murine VCAM-1/CD106 Fc chimera, following the bead manufacturer's instructions. As negative controls, beads were coated with bovine serum albumin (BSA, 0.1 mg/ml). The monoclonal antibodies were obtained from eBioscience (San Diego, CA), and VCAM-1 from R&D (Minneapolis, MN). Mice were pooled to provide 20×10^6^ purified CD4^+^ T cells per isolation. CD4^+^ T cells were incubated at 37^o^C for 2 minutes with the beads at a 1∶2 ratio of cells to beads. Stimulation was stopped with ice-cold PBS supplemented with protease and phosphatase inhibitor cocktails (Roche; Indianapolis, IN). Cells were lysed with a Branson Sonifier 450 (8 pulses at duty cycle = 10% and power output = 2). A magnet was used to separate the bead-captured material (IS fraction) from the non-IS fraction. The IS fraction was subsequently washed three times with PBS. Aliquots were removed from both fractions for western blot analysis, and samples snap-frozen at −80^o^C.

### Western blot analysis

Precast 4–15% gels and nitrocellulose were purchased from Biorad (Hercules, CA). Sonicated whole cell lysates equivalent to 1×10^5^ cells, and immune synapses from 1×10^6^ cells were loaded per well. Antibodies used were: anti-CD3ε (goat polyclonal), anti-Lck (rabbit polyclonal), anti-SLP 76 (rabbit polyclonal) and anti-VAV (rabbit polyclonal), all from Santa Cruz Biotechnology (Santa Cruz, CA). Anti-pLAT (rabbit polyclonal against tyrosine 191) was from Cell Signaling Technology (Danvers, MA).

### Sphingolipid analysis

Sphingolipids were analyzed by liquid chromatography, electrospray ionization tandem mass spectrometry (LC-ESI-MS/MS) with quantitation by comparison to spiked internal standards (purchased from Avanti Polar Lipids, Alabaster, AL) as previously described [15], [23]. Total sphingolipid contents were calculated by summing the amounts of the different N-acyl-chain-length subspecies. Molar fractions were calculated by normalizing the molar abundance of each species to the sum of all sphingolipids and dihydrosphingolipids in the corresponding sample. The number of biological replicates included for analysis is indicated in parenthesis above the individual bar graphs. Although monohexosylceramides were measured directly by this LC-ESI-MS/MS method, analysis of several of the samples by different conditions that resolved Glc-Cer and galactosylceramides [24], revealed that Glc-Cer accounted for the majority (85%) of monohexosylceramides in these samples (data not shown); thus, the term Glc-Cer is used throughout the text and figures. A method for analysis of gangliosides [25] was also applied to the samples, but the ganglioside signals for this number of CD4^+^ T cells were below the limit of detection (data not shown).

### Subspecies enrichment in CD4^+^ T cell immune synapses

Fold enrichment in the IS was calculated by dividing the molar fraction of each sphingolipid subspecies in the IS fraction by the corresponding molar fraction in the non-IS fraction. For the calculation of fold enrichment, molar fractions were calculated by normalizing the molar abundance of each sphingolipid species to the sum of all sphingolipids in the corresponding sample. Fold enrichments are not shown unless the molar abundance of a species in the non-IS fraction exceeds 2 pmol. This limits the appearance of artifacts due to low signal-to-noise in the denominator. A value of 1 indicates equivalent molar proportions in the IS and non-IS fractions. Values above 1 are indicative of IS enrichment. Values lower than 1 are indicative of poor retention in the IS fraction.

### CD4^+^ T cell proliferation experiments

Cell permeable, short-chain C2- and C6-Cer were obtained from Enzo Life Sciences (Plymouth Meeting, PA). Neutral sphingomyelinase, the serine palmitoyltransferase inhibitor myriocin, and the glucosylceramidase inhibitor conduritol B epoxide (CBE) were purchased from Sigma-Aldrich (St. Louis, MO). The glucosylceramide synthase inhibitor D,L-threo-1-phenyl-2-decanoylamino-3-morpholino-1-propanol (PDMP) was obtained from Cayman Chemical (Ann Arbor, MI). Anti-CD3ε antibody was from e-Bioscience (San Diego, CA), and anti-CD28 (no azide, low endotoxin) was purchased from BD pharmingen (San Diego, CA). For the assessment of T cell proliferation, 96-well flat-bottom plates (Nunc; Roskilde, Denmark) were coated with anti-CD3ε (5 µg/ml) for 90 minutes at 37°C and washed three times with PBS before cells were plated. To determine the effects of exogenous short-chain Cer, sphingomyelinase or inhibitors on T cell proliferation, triplicate wells each containing 1×10^5^ purified young or old murine CD4^+^ T cells were pre-incubated at 37°C for 2 hours with graded concentrations of these compounds in complete RPMI media and in 96-well round-bottom plates (Nunc). After two hours, cells were transferred to the plate-bound anti-CD3 plates and soluble anti-CD28 (2 µg/ml) antibody was added. Cells were then incubated for an additional 66 hours in the presence of Cer, sphingomyelinase, or inhibitors, and then 0.5 µCi/well of ^3^H-thymidine was added for an additional 6 hours to monitor CD4^+^ T cell proliferation. ^3^H-thymidine uptake was measured in a liquid scintillation counter (Beckman; Fullerton, CA). Data are expressed as absolute counts per minute (cpm).

### Determination of cell viability

CD4^+^ T cell viability was determined with the FITC annexin V apoptosis detection kit I from BD Pharmingen (San Diego, CA), following the manufacturer's instructions. Cells were analyzed on an Accuri C6 flow cytometer (Ann Arbor, MI).

### Statistical analysis

Data was analyzed for significant effects by Student's t test (unpaired, two-tailed, equal variance) using Systat statistical software. To determine if the addition of ceramides or inhibitors significantly affected CD4^+^ T cell proliferation or viability, the paired t test was used. Results are presented as means ± SEM or SD as indicated. Significance was set at p<0.05.

## Results

### Stimulation of CD4^+^ T cells with antibody-coated magnetic beads and separation into immune synapse (IS) and non-IS fractions

Although the formation of an immunological synapse between a T cell and an antigen-bearing APC is required for T cell activation, TCR-dependent signals are initiated within smaller structures distributed throughout the immunological synapse [26], [27], [28]. These structures, referred to as signaling microclusters, possess increased molar fractions of cholesterol, saturated phospholipid, and SM relative to the total T cell membrane; these properties are reminiscent of lipid rafts [22]. Therefore, it is plausible that age-related changes in the sphingolipid composition of the plasma membrane could influence T cell activation by altering this lipid microenvironment. However, the relevance of lipid rafts to T cell activation remains contentious [28], [29], [30].

**Figure 1:**
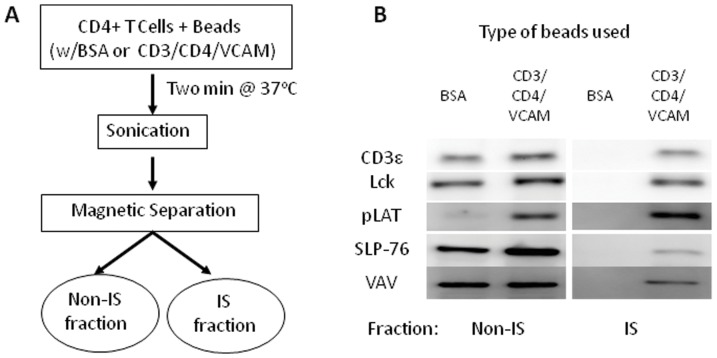
A schema for the stimulation of CD4^+^ T cells with antibody-coated magnetic beads and separation and characterization of the immune synapse (IS) and non-IS fractions. **A:** Purified murine CD4^+^ T cells were incubated for 2 min with control magnetic beads (BSA) or beads coated with anti-CD3ε, anti-CD4, and recombinant VCAM-1 (CD3/CD4/VCAM). Cells were then sonicated, and the IS and non-IS fractions separated using a magnet. **B:** Western blot analysis of the non-IS and IS fractions from control (BSA) or stimulated (CD3/CD4/VCAM) young T cells. The amount of sample (cell number equivalents) loaded on the IS lanes was 10 times that for the non-IS lanes. Shown is a representative blot from two independent experiments, each with n = 3 for total n = 6/age group.

We set out to identify age-related changes in the total sphingolipid content of T cells, and in the sphingolipid content of IS-restricted signaling microclusters. To this end, we purified CD4^+^ T cells by negative selection from the spleens of young (4–6 month-old) or old (24–26 month-old) C57BL/6 mice. The purified CD4^+^ T cells were incubated for two minutes with control or stimulatory magnetic beads, sonicated, and separated into two different fractions: the bead-bound immune synapse (IS) fraction and a bead-free non-IS fraction (Fig. 1A). Stimulatory beads were coupled with antibodies targeting CD3ε and CD4, and with VCAM-1, a VLA-4 ligand that enhances the retention of downstream signaling complexes at sites of TCR engagement [27]. Both stimulatory and control beads were blocked with bovine serum albumin.

Western blots showed that the stimulatory beads selectively retained the TCR and associated signaling proteins, including CD3ε, Lck, Y191-phosphorylated LAT (pLAT), SLP-76 and VAV (Fig. 1B). The presence of pLAT in non-IS sonicates from cells incubated with the stimulatory beads is diagnostic for T cell activation. Approximately 10 percent of the total CD3ε, Lck, and pLAT was associated with the beads, with SLP-76 and VAV showing weaker retention on the beads. These results parallel those reported with similar isolation procedures [26].

### Although most sphingolipid classes are increased in aged CD4^+^ T cells, Glc-Cer are reduced in aged CD4^+^ T cells

**Figure 2:**
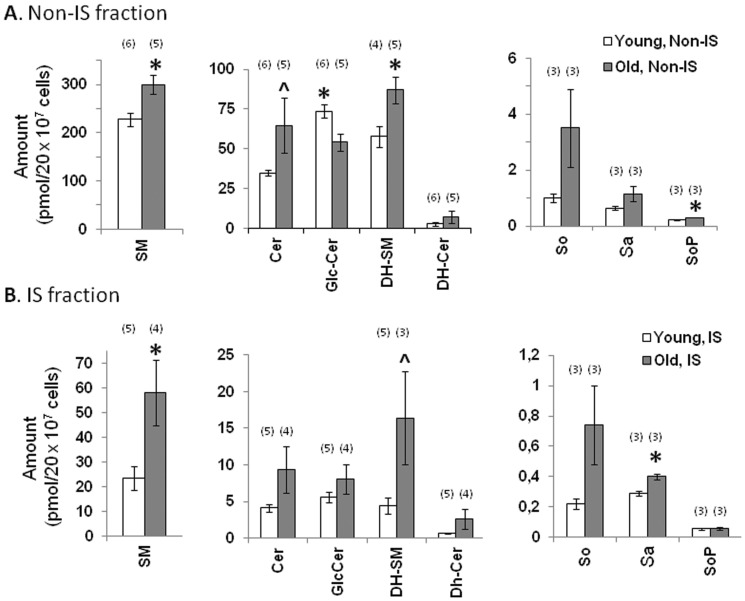
Although most sphingolipid classes are higher in aged CD4^+^ T cells, Glc-Cer are lower in aged CD4^+^ T cells. (**A**) Total amounts of main sphingolipid classes and long chain bases present in bead-depleted sonicates of bead-stimulated cells (non-IS fractions). White bars indicate results for young CD4^+^ T cells. Gray bars show results for old CD4^+^ T cells. Data is presented by sphingolipid category as the mean picomoles ± SEM detected in sonicates from 20×10^6^ cells. Two independent experiments were performed. In each experiment, three independent pools of T cells were prepared, stimulated, and sonicated. Each sonicate was subjected to sphingolipidomic analysis. The numbers in parenthesis above the bar graphs indicate the number of biological replicates included for the final analysis of each sphingolipid category and age group. *P* values were based on this number. (*p<0.05, ∧*p*<0.1 between age groups). (**B**). Total amounts of main sphingolipid classes and long chain bases present in CD4^+^ T cell IS fractions. White bars indicate results for young CD4^+^ T cells. Gray bars show results for old CD4^+^ T cells. Data is presented by sphingolipid category as the mean picomoles ± SEM detected in IS fractions from 20×10^6^ cells. These samples were prepared from the same pools used in figure 2A, and analyzed in parallel. The material analyzed here corresponds to the bead enriched (IS) fraction, whereas the material in figure 2A corresponds to the bead-depleted (non-IS) fraction. The numbers in parenthesis above the bar graphs indicate the number of biological replicates included for the final analysis of each sphingolipid category and age group. *P* values were based on this number. (*p<0.05, ∧*p*<0.1 between age groups).

Our non-IS fractions of primary CD4^+^ T cells are equivalent to bead-depleted sonicates of whole cells. Our analysis of the sphingolipid composition of these fractions indicates that SM are present at the highest concentrations, while Cer, Glc-Cer, and dihydrosphingomyelins (DH-SM) are present at intermediate levels, and long chain bases and downstream metabolites are present in extremely low amounts (Fig. 2A) (3). After applying correction factors based on cell volume and sample size, the total SM content was roughly comparable to that observed in a prior study of bead-stimulated Jurkat T cells [22]. However, the abundances of Cer and Glc-Cer relative to SM were considerably higher in our study of primary T cells. Relative to the young CD4^+^ T cells, most sphingolipids, long-chain bases, and SoP were elevated in old CD4^+^ T cells (Fig. 2A). Total SM and DH-SM were significantly higher in aged than in young CD4^+^ T cells (*p*<0.05). Per sample, SM levels in young CD4^+^ T cells were 227±14 pmol versus 299±20 pmol in the old group, an increase of 32% with age (mean ± SEM, *p*<0.05) (Fig. 2A). Likewise, DH-SM were 52% higher in old CD4^+^ T cells (*p*<0.05). Although the difference between Cer amounts in young and old CD4^+^ T cells did not reach statistical significance, there was a similar trend towards elevated quantities with age: Cer levels in young CD4^+^ T cell non-IS fractions were 34.8±1.8 pmol versus 64.8±17 pmol for the old group, a trend toward increase with age (mean ± SEM, *p*<0.1) (Fig. 2A). Although DH-SM, sphinganine (Sa) and sphingosine (So) were all numerically higher in the old group, none of these differences reached statistical significance. The end metabolite SoP was significantly higher in old CD4^+^ T cells (*p*<0.05). The unique exception to this pattern involved Glc-Cer, which were 35.6% less abundant in aged CD4^+^ T cells (73.6±4 pmol in young vs. 54.32±5.4 pmol in old, mean ± SEM, *p* = 0.02) (Fig. 2A). However, in the IS fractions, the abundance of Glc-Cer was similar between young and old, while the changes in other sphingolipid species largely followed the same direction though with different degree of significance compared to what were observed in non-IS fractions (Fig. 2B).

### The increased sphingolipid amounts observed in aged CD4^+^ T cell non-IS fractions are largely independent of fatty acyl chain length

The acyl chain length of sphingolipids can influence their structural and signaling properties [3], [31]. To determine whether alterations in the distribution of acyl chain lengths could contribute to age-related signaling defects in T cells, we compared the amounts of different sphingolipid subtypes in young and old CD4^+^ T cells. Although prior studies indicated that C16 SM was approximately 10-fold more abundant than the next most abundant SM subspecies in Jurkat T cells, our studies indicated that the distribution of sphingolipid subspecies in primary T cells is considerably more complex [22]. In particular, the longer-chain and unsaturated C24:1 subspecies of SM and DH-SM were co-dominant with the corresponding C16 subspecies, and appreciable amounts of the C18, C20, C22, and C24 subspecies were also present (Fig. S1). This data clearly indicates that sphingolipid acyl chain usage can vary widely within one cell type.

Since there are several ceramide synthase (CerS) enzymes that can attach fatty acyl chains of characteristic lengths to the Sa backbone to form DH-Cer, we reasoned that sphingolipids bearing specific acyl chains could be selectively altered with age [3], [9]. However, our results indicate that most sphingolipid subtypes are uniformly elevated in old CD4^+^ T cells (Fig. S1). Analysis of the different subspecies of SM indicated significantly higher amounts of C18, C20, C22, C24:1, C24, C26:1 and C26 subtypes in aged cells (*p*<0.05). DH-SM subspecies C14, C18, C20, C24:1 and C24 were also significantly elevated in the aged group (*p*<0.05) and a similar trend was found for C16 and C22 (*p*<0.1). Cer composition showed similar widespread subspecies elevations: old CD4+ T cells had higher levels of C18, C20, C22 and C24:1 subtypes (*p*<0.1), and significantly higher amounts of C24 and C26:1 (*p*<0.05). This could be the result of an increase in the output of the available CerS enzymes, or by a broad elevation in lipid content per cell in aged T cells.

We also examined the amounts of a recently discovered family of Cer, the 1-deoxydihydroceramides (deoxy-DH-Cer) and 1-deoxyceramides (deoxy-Cer), which are produced when alanine is used instead of serine by serine palmitoyltransferase [15]. These have been reported to bind to CD1b [16] and thus might also affect immune function in aging; however, these were only present in low amounts (less than 1% of ceramides), and did not differ between young and aged CD4^+^ T cells (Fig. S1).

A unique exception to this general pattern was found with Glc-Cer. When we compared the subspecies composition of the young and old groups, we found that the overall decrease in total Glc-Cer in aged CD4^+^ T cells was primarily due to the loss of the shorter chain-length C16, C18, C20, and C22 subspecies (Fig. S1, *p*<0.05); much smaller differences were observed with the longer chain-length C24:1, C24, C26:1, and C26 subtypes.

### The sphingolipid composition of the IS fraction is broadly similar to that of the non-IS fraction, with the exception of the Glc-Cer

To determine whether the sphingolipid composition of TCR-induced signaling microclusters is altered with age, we also examined purified immune synapse fractions that were captured using a modified immunoisolation procedure based on the work of Thomas Harder [26]. Briefly, T cells were incubated with stimulatory or control magnetic beads, allowed to form ‘synapses’, and sonicated in order to shear away all cellular material outside of the stimulatory microclusters. The resulting bead-bound IS fraction was recovered by magnetic isolation. In our hands, the fractional retention of SM in the IS fraction relative to the non-IS fraction of young CD4^+^ T cells was approximately 10 percent. This is considerably higher than the 1 percent fractional retention observed in prior studies employing Jurkat T cells [22]. This discrepancy could be due to the difference in cell type, the relative increase in surface-exposed membrane in the smaller primary cells, or to the inclusion of anti-CD4 and VCAM-1 on our stimulatory beads.

As shown in Fig. 2B, significantly higher quantities of SM were recovered from the immune synapses of old CD4^+^ T cells when compared to young (*p*<0.05). This includes both total and individual subspecies amounts. Per sample, the mean total SM recovered from young immune synapses was 23.36±4 pmol, compared to 58.17±13 pmol for the old group, an age-related increase of 149% (Fig. 2B). Most SM subspecies were significantly higher (*p*<0.05) in the aged CD4^+^ T cell IS fractions (Fig. S2). The average total DH-SM recovered from young immune synapses was 4.45±1 pmol, compared to 16.3±6 pmol for the old group, an age-related increase of 266% (*p*<0.1) (Fig. 2B). DH-SM subspecies C18, C20, C22 and C24:1 were significantly elevated in old IS fractions (*p*<0.05), and this trend was also seen for C14, C16, and C24 (*p*<0.1) (Fig. S2). Total Cer levels were higher in the old IS fractions (9.3±3 pmol) than in young (4.13±0.5 pmol), but this difference was not significant (Fig. 2B). However, the subspecies distribution showed a trend for higher C18, C20, C22, C24:1 and C24 in the aged fractions (*p*<0.1) (Fig. S2). Among the long chain bases, Sa was significantly elevated in old IS fractions (*p*<0.05) (Fig. 2B). In general, these findings are similar to those already described for the non-IS fractions (Figs. 2A and Fig. S1).

In contrast, the amounts of Glc-Cer recovered from CD4^+^ T cell immune synapses (Fig. 2B) was slightly higher in old (8.09±2 pmol/sample) than in young (5.6±0.74 pmol/sample), despite being significantly lower in the aged non-IS fractions (Fig. 2A). Although this difference did not reach statistical significance, there was a trend for higher levels of long-chain C24:1 and C24 subspecies in the old IS fractions (Fig. S2, *p*<0.1)). One possible explanation for this divergence from the pattern observed in the non-IS fraction is that the recovery of all sphingolipid subspecies is broadly elevated in the aged. If this is the case, then species that are proportionately underrepresented in the aged non-IS fractions may prove more abundant in the aged IS, in absolute terms, than the corresponding species in the young IS.

### The mole fraction of Glc-Cer relative to total sphingolipid is significantly reduced in both the non-IS and IS fractions derived from aged CD4^+^ T cells

**Figure 3:**
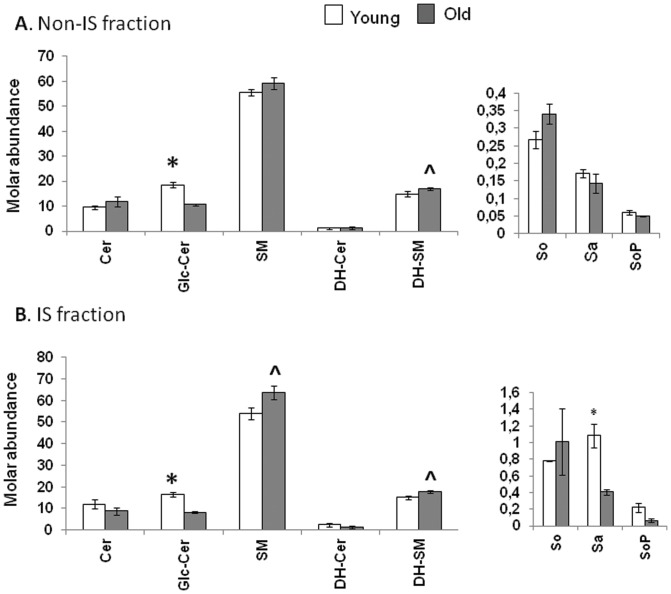
The mole fraction of Glc-Cer relative to total sphingolipid is significantly reduced in both the (A) non-IS and (B) IS fractions derived from aged CD4^+^ T cells. Bar graphs illustrating the relative abundance of sphingolipid classes in CD4^+^ T cell fractions, expressed as molar percents for each class in relation to the total sphingolipids and dihydrosphingolipids detected in each age group and fraction. The sphingolipid and dihydrosphingolipid picomol values reported in figures 2 were added to generate the total sphingolipid amounts for each age group and fraction. These values were then used as the basis to calculate the relative molar sphingolipid proportions, according to the formula:Molar % = (Sphingolipid Class in pmol)/((Total Sphingolipids in pmol) + (Total Dihydrosphingolipids in pmol)) ×100. (*p<0.05, ∧*p*<0.1 between age groups).

Since molar fractions are unaffected by differences in lipid abundance or capture efficiency, we searched for any sphingolipid class that showed age-dependent differences in enrichment in the non-IS or IS fractions. Fig. 3 plots the molar percent of each sphingolipid class to the total in each age group and fraction. Although the proportions of most sphingolipid classes were not significantly affected by fractionation, they did differ with age. In the non-IS fractions (Fig. 3A), Glc-Cer represented a larger molar percent of the total sphingolipid produced in young CD4^+^T cells (18.57±0.97%) when compared to old (10.6±0.48%). This represents a 43% decline with age (*p*<0.0001). Within immune synapses (Fig. 3B), Glc-Cer also represented a larger molar percent of the sphingolipids recovered from young CD4^+^ T cells (16.48±1.18%) when compared to old (8.27±0.33%). This represents a 50% decline with age (*p* = 0.0022). In contrast, the molar fraction of SM in the immune synapse increased 18% with age, from 53.89±2.7% to 63.64±3.2%, (mean ± SEM, *p* = 0.07) (Fig. 3B). Similarly, DH-SM were enriched in the aged non-IS (16.95±0.3%, *p* = 0.07) and IS (17.89±0.5%, *p* = 0.05) fractions, when compared to the corresponding young non-IS (14.91±0.9%) and IS (15.09±0.8%) fractions. The increased Cer and SM contents observed in the aged did not yield increased molar fractions because the old CD4^+^ T cells exhibited concomitant elevations in multiple sphingolipid classes, with the exception of the Glc-Cer.

**Figure 4:**
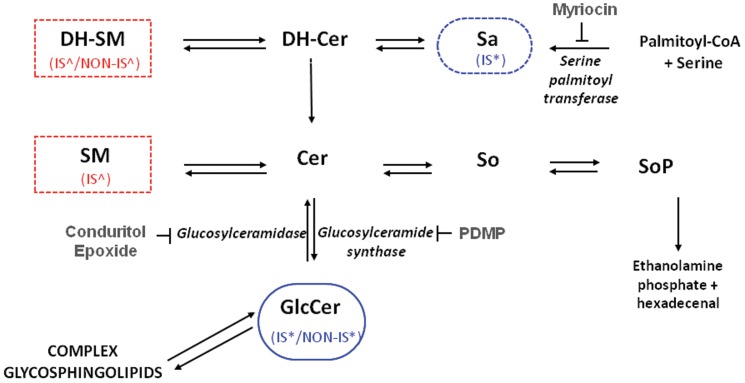
Schematic diagram of the sphingolipid metabolic pathway showing age-related molar fractional changes found in CD4^+^ T cell non-IS and IS fractions. Cer is considered the central hub of sphingolipid metabolism [3]. It is synthesized *de novo* in the endoplasmic reticulum by the condensation of serine and palmitoyl-CoA to form 3-keto-sphinganine (not shown), which is reduced to Sa. Sa is then N-acylated by a series of Cer synthase enzymes that display some selectivity towards particular fatty acyl chain lengths. The enzyme desaturase converts these DH-Cer into Cer, which are then transported to the Golgi apparatus. At this cellular subsite, Cer can be converted into a variety of interconnected sphingolipid products. Cer can be glycosylated to form Glc-Cer, followed by transformation into more complex GSL. Alternatively, Cer can be converted into SM, or follow a degradative pathway leading to So and SoP. The enzymes catalyzing these conversions are shown in italics, and the specificity of the three inhibitors used in the study (myriocin, CBE and PDMP) are illustrated. Sphingolipids that displayed significant (*p<0.05) age-related differences in their relative abundance in *both* the non-IS and IS fractions have a solid boundary. Sphingolipids that displayed a significant (*p<0.05) age-related difference or a trend (∧*p*<0.1) in at least one fraction have a dashed line. Legends (IS/NON-IS) indicate in which fraction(s) the difference was found. Blue indicates higher in young; red indicates higher in old.

Overall, our data identify three age-related changes in relative sphingolipid abundance. First, old CD4^+^ T cells show a statistically significant decline in Glc-Cer content that is apparent in both non-IS and IS fractions. Reciprocally, old CD4^+^ T cells exhibit a trend towards increased SM content in IS fractions. In addition, old CD4^+^ T cells exhibit a trend towards increased DH-SM content that is apparent in both non-IS and IS fractions. Fig. 4 is a schematic diagram of the sphingolipid metabolic pathway showing age-related changes in molar fractions found both in the non-IS and IS fractions.

### Subspecies analysis indicates that the reduced mole fraction of Glc-Cer in aged CD4^+^ T cells is driven by the loss of short chain Glc-Cer

**Figure 5:**
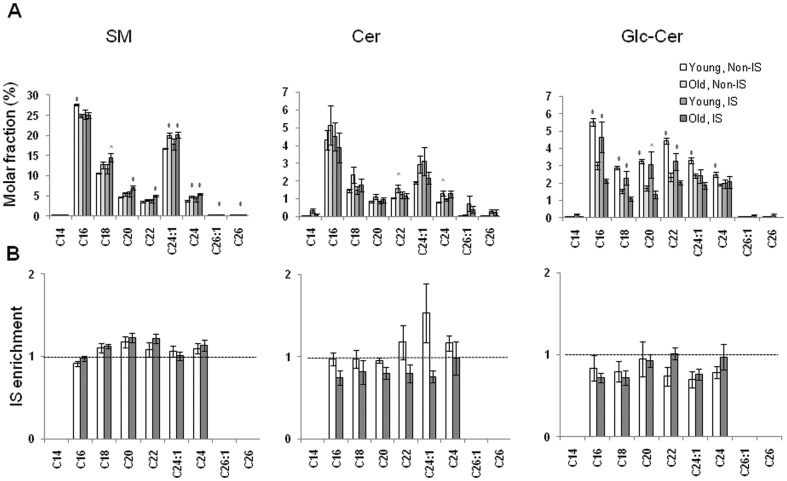
Subspecies analysis indicates that the reduced mole fraction of Glc-Cer in aged CD4^+^ T cells is driven by the loss of short chain Glc-Cer. (**A**) Relative abundance (molar fraction) of individual subspecies of SM, Cer, and Glc-Cer in young and old CD4^+^ T non-IS and IS fractions in relation to the total sphingolipid content (*p<0.05, ∧*p*<0.1 between age groups). (**B**) Relative enrichment of individual subspecies of SM, Cer, and Glc-Cer in young and old CD4^+^ T cell immune synapses in relation to the non-IS fractions. Fold enrichments are not shown unless the molar abundance of a species in the non-IS fraction exceeds 2 pmol. This limits the appearance of artifacts due to low signal-to-noise in the denominator. A dashed horizontal line indicates where this value is 1 (equivalent molar proportions in the IS and non-IS fractions). Values above 1 are indicative of IS enrichment. Values lower than 1 are indicative of poor retention in the IS fraction.

To examine the origin of the changes in the abundances of sphingolipid classes, we calculated the relative abundances of individual subspecies of SM, Cer, and Glc-Cer in relation to total conventional sphingolipids for both fractions and age groups (Fig. 5A). As seen in the total sphingolipid analysis, the C16 and C24:1 SM subspecies dominated, and appreciable amounts of C18, C20, C22, and C24 subspecies were present. A similar trend was observed for the Cer subspecies. Although significant differences in the fractional abundances of these species were observed with age, these differences were consistently small. Nevertheless, the SM and DH-SM subspecies observed in aged CD4^+^ T cells were consistently biased towards longer acyl chains, and away from C16 chains (Fig. 5A and data not shown). In addition, the reduced fractional abundances of Glc-Cer subspecies in aged CD4^+^ T cells were most pronounced in the short chain subspecies, and were less evident in the longest chain subspecies.

### Fractional enrichment of sphingolipid subspecies in CD4^+^ T cell immune synapses

To clarify whether CD4^+^ T cell immune synapses showed selective enrichment for individual subspecies of sphingolipids in relation to the corresponding non-IS fractions, we used the fractional abundances of each individual subspecies calculated above to generate relative enrichments (Fig. 5B). Most SM subspecies were equally distributed between the non-IS and immune synapse fractions, and no age-related differences were detected (Fig. 5B, left panel). Although three rare subspecies of Cer (C14, C26:1, and C26), as well as two subspecies of Glc-Cer (C14 and C26) appeared to show preferential enrichment within immune synapses, and this enrichment seemed more pronounced in young CD4^+^ T cells, this trend was not statistically significant and appeared to be driven by the poor signal-to-noise ratio resulting from the low abundances of these lipid subspecies (Figure 5B, middle and right panels).

### Both synthetic Cer and enzymatic Cer production inhibit CD4^+^ T cell proliferation

**Figure 6:**
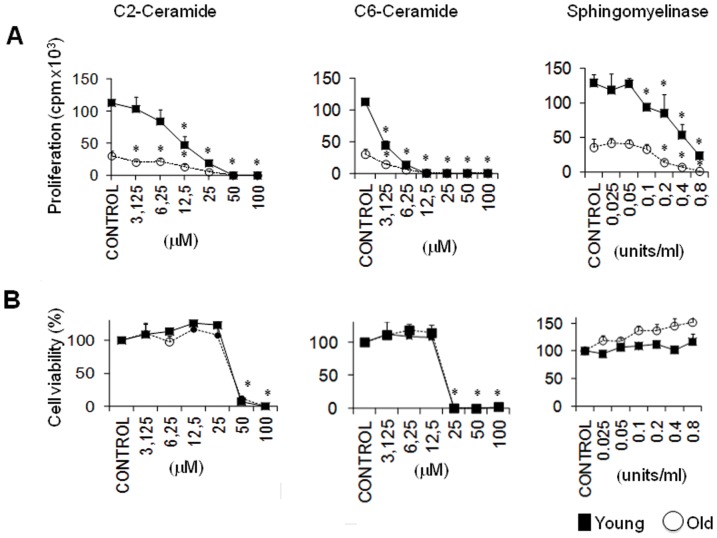
Both synthetic Cer and enzymatic Cer production inhibit CD4^+^ T cell proliferation. Young or old murine CD4^+^ T cells were pre-incubated with graded concentrations of the indicated compounds. After two hours, cells were activated in triplicate wells containing plate-bound anti-CD3 and soluble anti-CD28 antibodies, and incubated for another 72 hours in the presence of these compounds. (**A**). ^3^H-thymidine was added for the last 6 hours to monitor CD4^+^ T cell proliferation. Each data point represents mean counts per minute (CPM) ± SD of triplicate wells, each well containing 1×10^5^ purified young or old CD4^+^ T cells pooled from 3–5 mice. Asterisks indicate *p*<0.05 versus the corresponding untreated controls. The difference between the proliferation of control young and old CD4^+^ T cells is highly significant (*p*<0.001). One representative experiment of three is shown. (**B**). Cells treated as in (A) were tested for cell viability by staining with annexin V and propidium iodide (PI), prior to the addition of ^3^H-thymidine. Graphs illustrate the percent viable cells (PI negative, annexin V negative), after normalization to the untreated control samples, which were set to 100%. All statistics are as in (A).

Because total Cer levels are elevated in old relative to young CD4^+^ T cells, we investigated whether experimental manipulation of Cer levels could affect CD4^+^ T cell proliferation *in vitro*. We stimulated CD4^+^ T cells from young and old mice with plate-bound anti-CD3 and soluble anti-CD28 antibodies, in the presence or absence of the cell-permeable Cer, D-erythro-C2 Cer and D-erythro-C6 Cer (Fig. 6A). In addition, we used bacterial sphingomyelinase to convert sphingomyelin into Cer. All three approaches caused a dose-dependent reduction in CD4^+^ T cell proliferation (Fig. 6A). At concentrations up to 25 µM, the C2-Cer gradually decreased CD4^+^ T cell proliferation without reducing T cell viability (Figs. 6A and 6B, left panels). However, C2 Cer concentrations above 25 µM killed virtually all of the responding T cells. (Fig. 6B, left panel). Similar effects were observed with the C6-Cer, which inhibited proliferation at doses up to 12.5 µM, and caused cell death at higher doses (Figs. 6A and 6B, middle panels). Finally, bacterial sphingomyelinase significantly inhibited CD4^+^ T cell proliferation at concentrations between 0.1 and 0.8 U/ml, even though these doses did not significantly reduce the number of viable cells recovered (Figs. 6A and 6B, right panels). These data indicate that elevated Cer levels inhibit the proliferation of CD4^+^ T cells. Thus, age-related increases in Cer abundance could contribute to the proliferative defects observed in aged CD4^+^ T cells.

### Myriocin preferentially inhibits the proliferation of young CD4^+^ T cells without correcting the defective proliferation of aged CD4^+^ T cells

**Figure 7:**
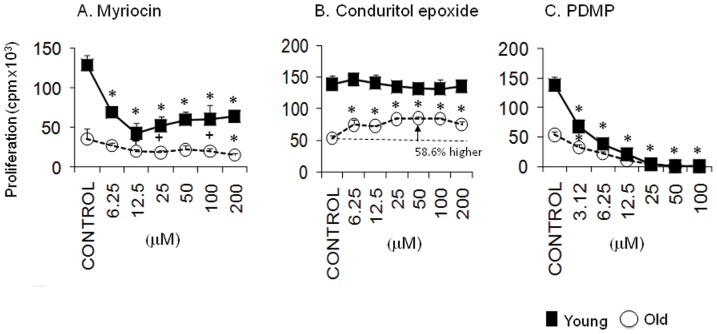
Inhibition of serine palmitoyl transferase activity with myriocin preferentially inhibits the proliferation of young CD4^+^ T cells, but suppression of Glc-Cer hydrolysis preferentially enhances the proliferation of Glc-Cer-poor aged CD4^+^ T cells. Young and old murine CD4^+^ T cells were treated with the indicated concentrations of (A) myriocin, (B) conduritol B epoxide (an inhibitor of glucosylceramide hydrolysis) or (C) PDMP (an inhibitor of glucosylceramide synthesis) and stimulated as in Figure 6. Proliferation assays and all statistical analyses were performed as in Figure 6A. Asterisks and plus signs respectively indicate *p*<0.05 and *p*<0.1 versus the corresponding untreated controls. The difference between the proliferation of control young and old CD4^+^ T cells is highly significant (*p*<0.001). One representative experiment of two is shown.

To determine if the defective proliferation of aged CD4^+^ T cells can be corrected by reducing the overproduction of sphingolipids, we inhibited *de novo* sphingolipid synthesis with myriocin (Fig. 7). Previous studies have shown that myriocin suppresses the proliferation of the murine cytotoxic cell line CTLL-2 with an IC_50_ of 15 nM [32], [33]. Furthermore, the inhibition of sphingolipid synthesis by myriocin also impedes the activation and proliferation of human T lymphocytes [34]. This compound inhibited the proliferation of both young and old CD4^+^ T cells at doses between 12.5 and 200 nM (Fig. 7). However, this compound preferentially inhibited the proliferation of young CD4^+^ T cells at doses between 6.25 and 100 nM, and only achieved statistically significant reductions in the proliferation of young T cells within this range. Although the inhibition of *de novo* synthesis of sphingolipids with low doses of myriocin failed to correct the defective proliferation of aged CD4^+^ T cells, these doses profoundly reduced the proliferation of young T cells. Thus, the high levels of proliferation observed in young T cells appear to be far more dependent on *de novo* sphingolipid synthesis than the low levels of proliferation observed in the old T cells.

### Suppression of Glc-Cer hydrolysis preferentially enhances the proliferation of Glc-Cer-poor aged CD4^+^ T cells

Although most sphingolipid metabolites were elevated in aged CD4^+^ T cells, Glc-Cer were uniquely less abundant in these cells (Fig. 2). Furthermore, the molar fraction of Glc-Cer was reduced by approximately 50 percent in in aged T cells (Fig. 3). To test whether this difference contributes to the proliferative defects observed in aged T cells, we employed specific inhibitors of the enzymes that either synthezise or degrade Glc-Cer. CBE is a specific, irreversible inhibitor of glucosylceramidase (Fig. 4), and should increase Glc-Cer content [13]. In fact, CBE partially corrected the proliferative defect observed in aged CD4^+^ T cells, significantly increasing the proliferation of these cells, up to 58.6% above control levels (Fig. 7). Young CD4^+^ T cells were less affected by CBE, and only showed a 5.8% increase in proliferation. Thus, the defect in Glc-Cer content may contribute to the proliferative defects observed in aged T cells. In contrast to CBE, PDMP is an inhibitor of Glc-Cer synthase [13], [35]. Consistent with the above hypothesis PDMP, which should decrease Glc-Cer content, strongly inhibited the proliferation of both young and old CD4^+^ T cell (Fig. 7). These data indicate that young and old T cells both depend on Glc-Cer for proliferation, and that the proliferative defect in old T cells may be related to a deficiency in Glc-Cer.

## Discussion

In this study, we analyzed the sphingolipid composition of young and old CD4^+^ T cells. Our investigation uncovered significant perturbations in the sphingolipid composition of aged CD4^+^ T cells. With the exception of Glc-Cer, most sphingolipid metabolites were more abundant in aged CD4^+^ T cells. In contrast, the absolute and fractional abundances of Glc-Cer declined with age. Finally, SM, DH-SM, and Glc-Cer subspecies were biased towards greater acyl chain lengths with age.

The proximate causes of these age-dependent shifts in sphingolipid distribution are not well understood. However, the increased sphingolipid abundance observed in the aged is unlikely to be caused by an age-related increase in cellular lipid content, as this would be expected to concomitantly increase Glc-Cer abundance. Consequently, the increased pool of sphingolipid observed in aged CD4^+^ T cells is likely to reflect either increased *de novo* synthesis or reduced sphingolipid turnover. Similarly, the decreased abundances of Glc-Cer in aged T cells might arise from specific decreases in the activity of Glc-Cer synthetase or increases in the activity of glucosylceramidase; however, there might also be contributions from differences in the intracellular trafficking of these compounds by proteins such as CERT and FAPP2 and/or biosynthesis of downstream complex GSL, such as gangliosides, from Glc-Cer. Finally, the age-dependent-bias towards increased acyl chain length could reflect a relative reduction in the activity of the C16-specific Cer synthetase, CerS5, or relative increases in the activities of long-chain specific Cer synthetases, such as CerS1, CerS2, and CerS4 [9]. If validated, these observations could provide insights into the molecular basis of age-related changes in T cell function.

Although the underlying causes of these age-dependent perturbations in sphingolipid metabolism are not known, several processes associated with aging can impinge on sphingolipid metabolism. For example, telomere shortening is accelerated in C57BL/6 mice when they reach the age of 24 months [36], [37]. This produces genotoxic stresses that provoke widespread p53 activation [38]. Since Cer is commonly produced downstream of p53, it is possible that age-dependent telomere shortening indirectly mediates the observed changes in sphingolipid metabolism [6]. However, it is unclear whether the p53-dependent elevation in Cer levels results from increased *de novo* sphingolipid synthesis, or whether a different pathway, such as sphingomyelinase activation, is the primary driver of Cer production in response to p53 activation. Aging is also associated with suboptimal mitochondrial function, which leads to increased reactive oxygen species (ROS) production. Increases in ROS production can activate sphingomyelinases, which may contribute to increased Cer levels in aged T cells [39]. Similarly, reducing conditions inhibit DH-Cer desaturase, a crucial enzyme involved in the synthesis of Cer, providing another route by which elevated ROS production could contribute to Cer production in aged CD4^+^ T cells [40].

*In vitro*, moderate elevations of Cer content inhibited CD4^+^ T cell proliferation without impacting cellular viability. These results are consistent with reports showing that Cer-dependent pathways can lead to cell cycle arrest. For example, exogenous C6-Cer significantly inhibited the cyclin dependent kinase CDK2, a master regulator of cell cycle progression [8]. Cer inhibited CDK2 by inducing the CDK inhibitor p21, and by activating the protein phosphatases PP1 and PP2A, which negatively regulate CDK2 [8]. This effect was observed at a C6-Cer concentrations comparable to those used in our studies (3–10 µM). Thus, the increased Cer levels in old CD4^+^ T cells could contribute to the reduced proliferative responses of these cells. However, the Cer-induced inhibition of T cell proliferation did not differentially impact young and old T cells.

Glc-Cer were both less abundant and represented a lower relative proportion of the sphingolipids in old CD4^+^ T cells. Interestingly, experimental enhancement of Glc-Cer levels with the irreversible inhibitor conduritol B epoxide preferentially enhanced the proliferation of aged CD4^+^ T cells, partially correcting the defect seen in the aged group. In contrast, reducing Glc-Cer levels with the inhibitor PDMP inhibited CD4^+^ T cell proliferation (Fig. 7). The reciprocal effects of these compounds on cellular proliferation are not unique to CD4^+^ T cells, but appear to constitute a “rheostat” that controls cell cycle progression in other systems as well. In this regard, enhanced Glc-Cer synthesis has been linked to the pathological hyperproliferation of renal cell in polycystic kidney disease [12]. In addition, cancer cells that synthesize potentially toxic levels of Cer as a result of chemotherapeutic insults often respond by shunting sphingolipid metabolism towards enhanced Glc-Cer synthesis [41]. The functional outcome is increased multidrug resistance. This suggests that enhanced synthesis of Glc-Cer can in some instances override the cytostatic effects of ceramides. In support of our findings, Zhu et al. [14] recently showed that lowering GSL in CD4^+^ T cells significantly lowered IL-2 production and T cell proliferation in Jurkat and primary human T cells. The partial correction of age-associated defects in CD4^+^ T cell *in vitro* proliferation by conduritol B epoxide suggests that it might be useful in an *in vivo* setting. Although chronic administration of this compound in mice has secondary effects [42], it is possible that it might “boost” the responses of aged mice.

The molecular mechanisms involved in Glc-Cer-dependent cell cycle modulation remain obscure. Importantly, it is unclear whether these effects are mediated by Glc-Cer themselves, or by their more complex GSL derivates. As noted above, a metabolic shunt capable of transforming Cer into Glc-Cer could promote proliferation by lowering Cer levels. Alternatively, complex GSL such as gangliosides, may participate directly in lectin-mediated complexes with transmembrane or GPI-linked receptors. In this regard, gangliosides have been shown to modulate signaling by several growth factor receptors, such as the epidermal growth factor, fibroblast growth factor, platelet-derived growth factor, and insulin receptors [43]. In CD4^+^ T cells, the ganglioside GM3 has been shown to co-precipitate with Zap-70 upon T cell activation [44], and CD4 ligation has been shown to induce the aggregation of the gangliosides GM1 and GM3 [45]. Thus, it is possible that the increased Glc-Cer levels in young CD4+ T cells reflect the presence of structurally complex GSL that are capable of contributing positively to early TCR signaling events.

The impact of sphingolipid composition on membrane microdomain structure may also contribute to age-related defects in CD4^+^ T cell proliferation [28]. TCR activation triggers the recruitment of signaling proteins and adaptors into signaling microclusters, which prior studies have shown to be enriched in cholesterol, SM and saturated phosphatidylcholine species [22]. With age, we observed increases in the absolute abundances of most sphingolipids, decreases in the fractional abundances of Glc-Cer, and a bias towards the usage of longer acyl chains in SM and Glc-Cer. Thus, alterations in the sphingolipid composition of aged T cells may directly impact the assembly of these signaling structures by altering the structural properties of the raft-like membrane domains surrounding the activated TCR. Furthermore, there are precedents for the direct modulation of protein trafficking and conformation by sphingolipid metabolites [46], [47]. Since T cell activation involves changes in protein trafficking and conformation, age-related changes in the availability of sphingolipid metabolites could also regulate these events.

We also examined the sphingolipid content of ‘immune synapses’ isolated from primary murine T cells using stimulatory magnetic beads. This ‘IS’ fraction captured approximately 10 percent of the sphingolipids present in non-IS fractions. Furthermore, the IS fraction recapitulated the changes in sphingolipid abundance observed in aged T cells, and did not show any dramatic shifts in the fractional adundances of sphingolipid subspecies relative to the non-IS fractions. Employing a similar immunoisolation methodology, Zech, *et al.* recently investigated the distribution of lipids in signaling microdomains derived from Jurkat T cells [22]. This study confirmed that the IS fraction was similar in composition to lipid rafts, insofar as it was highly enriched in cholesterol and SM relative to phosphatidylcholine. However, the IS fraction examined by Zech *et al.* captured only 1 percent of the SM found in intact cells, and the fractional abundance of SM in the IS was increased relative to Cer and hexosylceramide. These divergent observations could be due to differences in the cell types studied or the methodologies employed. For example, our data indicate that there are qualitative differences in the acyl chain usage of SM in primary murine T cells and Jurkat T cells; these differences may drive changes in the lipid composition of the IS. Alternately, the increased retention of SM in our study may be a trivial consequence of the smaller size of primary T cells, which may expose a higher fraction of SM on the plasma membrane, where it would be available to participate in the IS. Finally, the increased SM retention observed in our study may be due to our inclusion of stimulatory ligands that were not present in the Zech study, *e.g.* anti-CD4 and VCAM-1.

In conclusion, CD4^+^ T cells exhibit age-dependent perturbations in their sphingolipid composition. These perturbations have the potential to negatively modulate proliferation responses. Nevertheless, the modulation of Glc-Cer metabolism overcame some of these inhibitory effects, suggesting an experimental intervention strategy for improving immune function in the aged. Of note, some of the sphingolipid perturbations described in cancer or polycystic kidney disease cells in association with uncontrolled proliferation appear to be the exact opposite of those associated with hypoproliferation in aged T cells. Even primitive eukaryotes like the parasite *Giardia lamblia* appear to rely on this pathway [48]. This underscores the universal conservation and importance of these pathways for the control of cell cycle progression.

## Supporting Information

Figure S1 **The increased sphingolipid abundances observed in aged CD4^+^ T cell non-IS fractions are largely independent of fatty acyl chain length.** Individual subspecies amounts present in bead-depleted sonicates (non-IS fractions) prepared from equivalent numbers (20×10^6^) of purified young and old murine CD4^+^ T cells. White bars indicate results for young CD4^+^ T cells. Gray bars show results for old CD4^+^ T cells. The data represent mean picomoles ± SEM of each sphingolipid subspecies detected in the two independent experiments shown in Figure 2A. (*p<0.05, ∧*p*<0.1 between age groups).
(TIF)

Figure S2 **The increased sphingolipid abundances observed in aged CD4^+^ T cell IS fractions are largely independent of fatty acyl chain length.** Individual subspecies amounts present in bead-enriched (IS) fractions prepared from equivalent numbers (20×10^6^) of purified young and old murine CD4^+^ T cells. White bars indicate results for young CD4^+^ T cells. Gray bars show results for old CD4^+^ T cells. The data represent mean picomoles ± SEM of each sphingolipid subspecies detected in the two independent experiments shown in Figure 2B. (*p<0.05, ∧*p*<0.1 between age groups).
(TIF)
